# External Ventricular Drainage in Patients With Acute Aneurysmal Subarachnoid Hemorrhage After Microsurgical Clipping: Our 2006-2018 Experience and a Literature Review

**DOI:** 10.7759/cureus.12951

**Published:** 2021-01-27

**Authors:** Anton Konovalov, Oleg Shekhtman, Yury Pilipenko, Dmitry Okishev, Olga Ershova, Andrey Oshorov, Arevik Abramyan, Irina Kurzakova, Shalva Eliava

**Affiliations:** 1 Vascular Surgery, Burdenko Neurosurgical Center, Moscow, RUS; 2 Neurosurgery, Burdenko National Medical Research Center of Neurosurgery, Moscow, RUS; 3 Vascular Surgery, Burdenko National Medical Research Center of Neurosurgery, Moscow, RUS; 4 Epidemiology and Public Health, Burdenko National Medical Research Center of Neurosurgery, Moscow, RUS; 5 Internal Medicine: Critical Care, Burdenko National Medical Research Center of Neurosurgery, Moscow, RUS

**Keywords:** external ventricular drain, evd, sah, aneurysm rupture

## Abstract

Introduction

The placement of an external ventricular drain (EVD) is widely practiced in neurosurgery for various diseases and conditions accompanied by impaired cerebrospinal fluid (CSF) circulation, intracranial hypertension (ICHyp), intraventricular hemorrhage (IVH), and hydrocephalus. Specialists have been using this method in patients with acute aneurysmal subarachnoid hemorrhage (aSAH) for more than 50 years. Extensive experience gained at the Burdenko Neurosurgical Center (BNC) in Moscow, the Russian Federation, in the surgical treatment of patients with acute aSAH enabled us to describe the results of using an EVD in patients after microsurgery. The objective of the research was to assess the effectiveness and safety of the EVD and clarify the indications for the microsurgical treatment of aneurysms in patients with acute SAH.

Materials and methods

From 2006 until the end of 2018, 645 patients registered in the BNC database underwent microsurgery for acute (0-21 days) aSAH. During the case study, we assessed the severity of hemorrhage according to the Fisher scale, the condition of patients on the Hunt-Hess (H-H) scale during surgery, the time of placement of EVD (before, during, and after surgery), and the duration of EVD. The number of patients with parenchymal intracranial pressure (ICP) transducers was assessed by the degree of correlation of ICP data through the EVD and parenchymal ICP transducer. One of the aims of the research was to compare the frequency of using EVD and decompressive craniectomy (DCH). The incidence of EVD-associated meningitis was analyzed. The need for a ventriculoperitoneal shunt (VPS) in patients after using EVD was also assessed. Overall outcomes were assessed using a modified Rankin scale (mRS) at the time of patient discharge. Exclusion criteria were as follows: patients aged less than 18 years and the lack of assessed data. Patients undergoing endovascular and conservative treatments also were excluded.

Results

Among the patients enrolled in the study, 22% (n=142) had EVD. Among these, 99 cases (69.7%) had EVD installed in the operating room just before the start of the surgical intervention. In some cases, ventriculostomy was performed on a delayed basis (16.3%). A satisfactory outcome (mRS scores of 1 and 2) was observed in 24.7% (n=35). Moderate and profound disability at the time of discharge was noted in 55.7% (n=79). Vegetative outcome at discharge was noted in 8.4% (n=12), and mortality occurred in 12.3% (n=15).

Conclusion

EVD ensures effective monitoring and reduction of ICP. EVD is associated with a relatively low risk of infectious, liquorodynamic, and hemorrhagic complications and does not worsen outcomes when used in patients with aSAH. We propose that all patients in the acute stage of SAH with H-H severity of III-V should receive EVD immediately before surgery.

## Introduction

Neurosurgeons have widely used the placement of an external ventricular drain (EVD) for various diseases and conditions accompanied by impaired cerebrospinal fluid (CSF) circulation, intracranial hypertension (ICHyp), intraventricular hemorrhage (IVH), and hydrocephalus for decades. Specialists have been using this method in patients with acute aneurysmal subarachnoid hemorrhage (aSAH) for more than 50 years. It is used for acute occlusive hydrocephalus to reduce and control intracranial pressure (ICP) and to sanitize the CSF in cases of IVH [1,2]. In subacute SAH, external drainage is used to prevent resorptive hydrocephalus, as well as to assess the need to install a shunt system [3].

Even though EVD is included in all kinds of management and treatment protocols for patients with acute SAH, relatively few published studies have assessed its role with regard to treatment outcomes; this is surprising given both its positive effect and the frequency of various complications. One of the first publications on the topic was the paper by Kusske et al. in 1973. As per this study, after the insertion of EVD, a significant improvement in the patient’s clinical condition was noted against the background of post-hemorrhagic hydrocephalus [4]. Subsequently, the problem was discussed in several studies; however, the indications for EVD, the duration of drainage, and the prevention of potential complications still remain topics of debate [5-8].

Extensive experience gained at the Burdenko Neurosurgical Center (BNC) in the surgical treatment of patients with acute aSAH enabled us to discuss the results of using an EVD in patients after microsurgery. The objective of the research was to analyze the use of this option in patients after microsurgical treatment of aneurysms in acute SAH.

## Materials and methods

From 2006 until the end of 2018, 645 patients registered in the BNC database underwent microsurgery for acute (0-21 days) aSAH. Inclusion criteria were as follows: adult patients who had a microsurgical clipping of intracranial aneurysm during the acute stage of SAH and underwent EVD installation before, during, or after surgery. The study group consisted of 142 cases (Table 1). During the study, we assessed the severity of hemorrhage according to the Fisher scale, the condition of patients on the Hunt-Hess (H-H) scale during surgery, the time of placement of EVD (before, during, and after surgery), and the duration of EVD. The number of patients with parenchymal ICP transducers was assessed by the degree of correlation of ICP data through the EVD and parenchymal ICP transducer. One of the aims of the research was to compare the frequency of using EVD and decompressive craniectomy (DCH). The incidence of EVD-associated meningitis was analyzed. The need for a ventriculoperitoneal shunt (VPS) in patients after using EVD was also assessed. Overall outcomes were assessed using a modified Rankin scale (mRS) at the time of patient discharge. Exclusion criteria were as follows: patients aged less than 18 years and the lack of assessed data. Patients undergoing endovascular and conservative treatments also were excluded.

**Table 1:** Patient characteristics EVD: external ventricular drain; SAH: subarachnoid hemorrhage; TCD: transcranial Doppler; SD: standard deviation

Variables	Values
Total number of patients	142
Age (years)	Range: 18-73; mean: 51.4
Sex, n (%)	Male: 66 (46.5%); female: 76 (53.5%)
Localization of the aneurysm	
Anterior communicating artery (AComA), n (%)	68 (47.9%)
Internal carotid artery (ICA), n (%)	32 (22.5%)
Middle cerebral artery (MCA), n (%)	25 (17.6%)
Pericallosal artery, n (%)	8 (5.6%)
Posterior cerebral artery (PCA), n (%)	4 (2.8%)
Posterior inferior cerebellar artery (PICA), n (%)	5 (3.6%)
The period of EVD implantation after SAH	
0-3 days, n (%)	53 (37.3%)
4-7 days, n (%)	46 (32.4%)
8-14 days, n (%)	25 (17.6%)
15-21 days, n (%)	18 (12.7%)
Hunt-Hess severity scale	
I, n (%)	1 (0.8%)
II, n (%)	19 (13.4%)
III, n (%)	63 (44.4%)
IV, n (%)	44 (30.9%)
V, n (%)	15 (10.5%)
Fisher scale	
Grade 2, n (%)	6 (4.2%)
Grade 3, n (%)	58 (40.8%)
Grade 4, n (%)	78 (54.9%)
Severe cerebral spasm (more than 240 cm/sec according to TCD), n (%)	37 (26.1%)
The period of the installation of EVD in relation to surgical treatment	
Simultaneously, n (%)	104 (73.2%)
Before, n (%)	2 (1.4%)
After, n (%)	36 (25.3%)
Duration of EVD (days)	Range: 3-14; mean ±SD: 7.3 ±2.2
Ventricular peritoneal shunt, n (%)	17 (11.9%)
Decompressive hemicraniectomy, n (%)	29 (20.4%)
Meningitis, n (%)	18 (12.6%)
Outcomes (modified Rankin scale score)	
1 (asymptomatic), n (%)	12 (8.5%)
2 (light disability), n (%)	23 (16.2%)
3 (moderate disability), n (%)	49 (34.5%)
4 (severe disability), n (%)	30 (21.2%)
5 (vegetative state), n (%)	12 (8.4%)
6 (death), n (%)	16 (11.2%)

EVD installation technique

**Figure 1:**
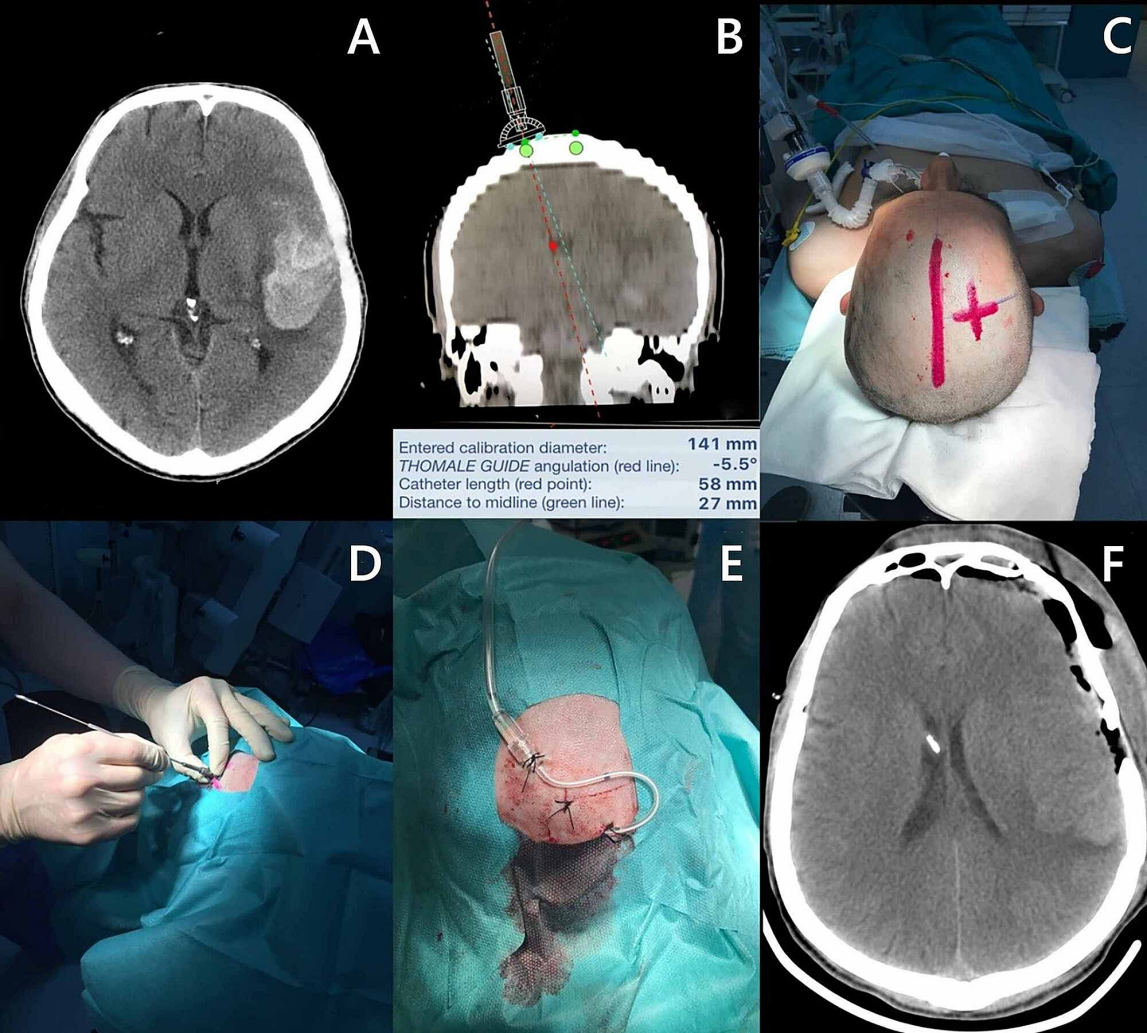
Installation of EVD The images show the various stages in the installation of an EVD into the anterior horn of the right lateral ventricle in a patient on the fourth day after SAH from the left MCA aneurysm using a navigation device A: axial CT of the head at admission. Dislocation of the midline structures of 2 mm and an intracerebral hematoma with a volume of 30 ml were observed; B: calculation of parameters for planning the EVD navigation unit using the Thomale software; C: application of measurements and selection of the trephination point for the installation of EVD; D: installation of EVD as per the Thomale guide; E: the type of postoperative wound; F: CT of the head after surgery EVD: external ventricular drain; SAH: subarachnoid hemorrhage; MCA: middle cerebral artery; CT: computed tomography

The EVD was installed using the accepted standard technique through the standard Kocher’s point. For narrow or constricted ventricles, navigation devices were used (Figure 1) [8]. The distal end of the drainage tube was passed under the skin and brought out 3-5 cm from the initial incision through a counter-aperture incision. This manipulation serves as a measure of infection prevention. The CSF was drained through a closed sterile system into a plastic reservoir. The reservoir was fixed at or above the level of the external auditory canal to avoid overdraining. This position could then be changed depending on the level of ICP. In the absence of clinical signs of infection, a CSF analysis was performed every two to four days. The duration of the drainage was determined based on several factors: clinical necessity, the appearance of signs of meningitis, and cessation of functioning (thrombosis of the drainage tube, technical problems). The drainage lasted 3-14 days (mean: 7.3 ±2.2 days).

EVD and ICP monitoring

**Figure 2:**
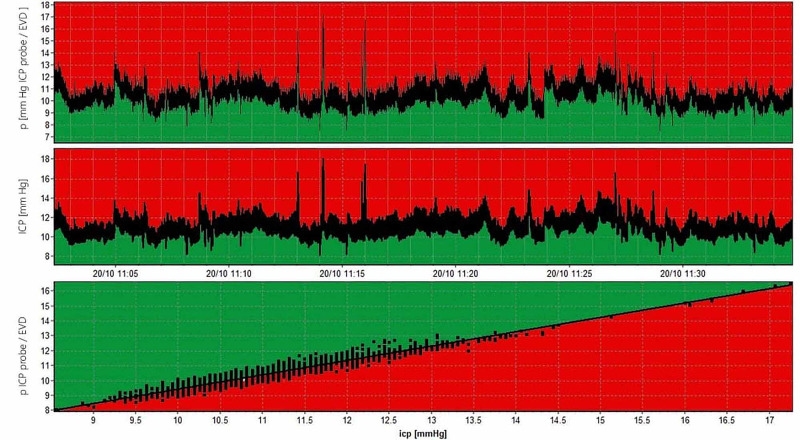
Correlation between parenchymal ICP probe and EVD R=0.96 ICP: intracranial pressure; EVD: external ventricular drain

Intraparenchymal ICP monitoring was not performed in all cases. Thus, the number of intraparenchymal ICP sensors was 50 (35%). The average period of ICP monitoring was 6 ±1.7 days (range: 4-10 days). In three patients, we were able to compare and evaluate the ICP monitoring data using an intraparenchymal sensor and an EVD, and to assess the degree of correlation of the obtained data (Figure 2). The quality of measurement through EVD depends on the permeability of the drainage, the localization of the measuring sensor, the quality of recalibration (every 8-12 hours), external interference, manipulations with the head end of the bed, as well as CSF drainage. The best way to exclude errors when comparing ICP parameters from the sensor and EVD is to compare the ICP pulse wave (morphology and amplitude). Thus, it can be noted that the measurement with the EVD is comparable with the data of direct invasive control.

## Results

Among the patients enrolled in the study, 22% (142/645) had EVD. Among these, 99 cases (69.7%) had EVD installed in the operating room just before the start of the surgical intervention. In some cases, ventriculostomy was performed on a delayed basis (16.3%). In the early period, EVD was installed in two patients in extremely severe condition (1.6%) before surgery, after which the condition of the patients improved and the microsurgical clipping of the aneurysm was performed on the next day.

EVD was installed in 20 (14.1%) patients with H-H severity of I-II. In all cases, the condition of patients worsened after the surgical treatment, which required the implantation of EVD during or after the operation. Out of 13 patients with intraoperative complications, seven (54%) developed intraoperative rupture, and six (46%) cases had EVD installed as per the decision of the operating surgeon based on their intraoperative findings. In four patients (20%), the spasm was treated with intra-arterial injection of verapamil. Three patients (15%) developed clinically significant hydrocephalus, which required the implantation of EVD, followed by a planned VPS. A larger number of patients with drains had H-H severity of III (44.4%) and IV (30.9%), which was associated with more active surgical management in these groups. In extremely severe patients, the drain was installed in 15 (10.5%) cases.

ICHyp was often an indication for EVD installation. The fight against this complication is one of the most important problems in the treatment of acute patients, as edema and dislocation of the brain, due to various reasons, remain the main cause of death. Installation of EVD is not the only element of treatment for ICHyp, but one of the most effective. However, DCH is also referred to as the “surgical” method. In our cohort, DCH was performed in 27 patients (22.1%).

**Figure 3:**
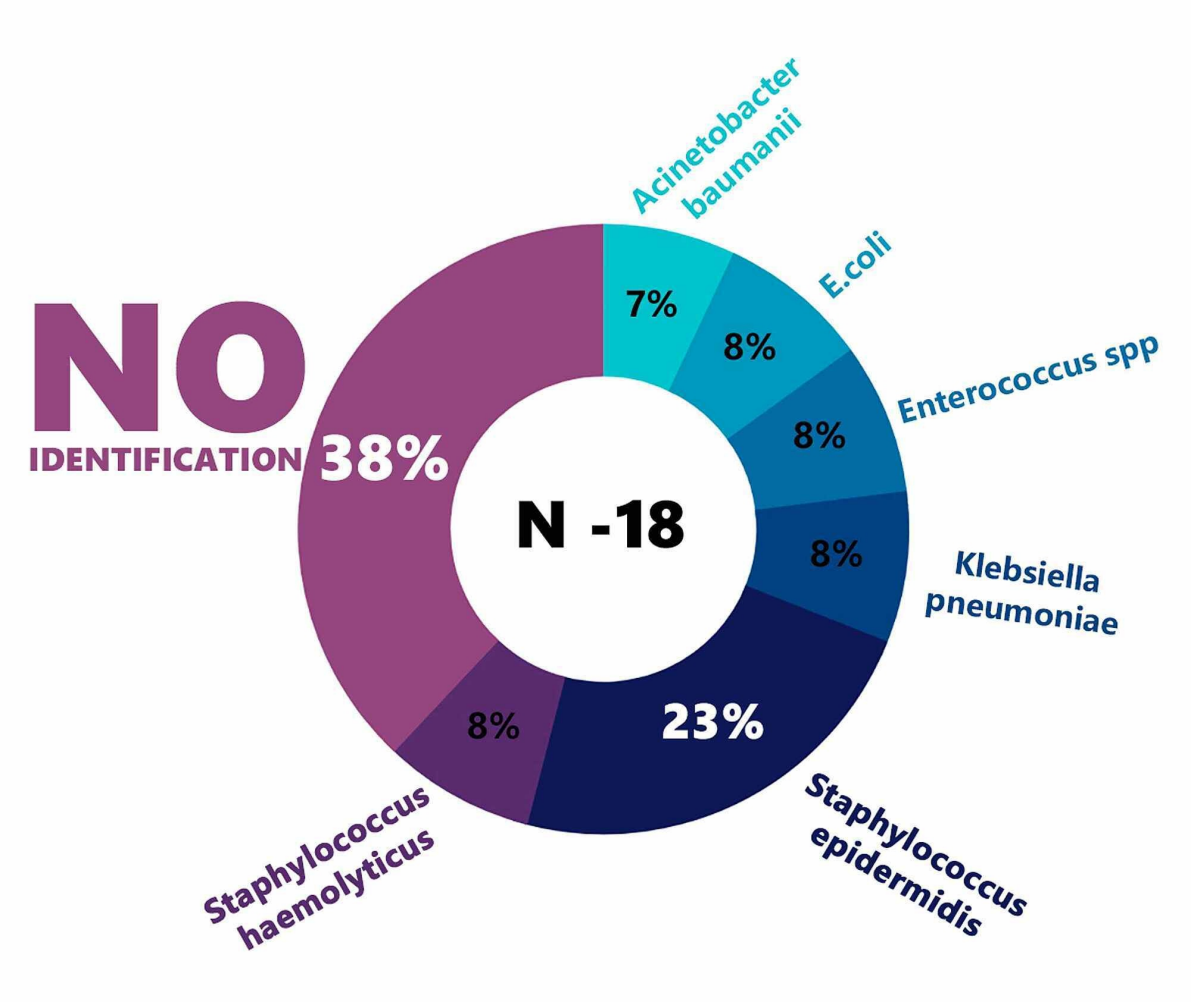
Data related to EVD-associated meningitis pathogens for the period 2006-2018 EVD: external ventricular drain

Drainage-associated meningitis was diagnosed in 18 patients, which accounted for 12.6% of all patients with EVD (Figure 3). Four of them died directly from meningitis. Microbiological examination revealed the pathogen in only 61.1% (11 of 18 patients). At the same time, changes in the structure of pathogens - meningitis caused by *Acinetobacter baumannii*, occurred significantly less; various *Staphylococci* account for a high percentage of EVD-associated meningitis; the most common cause of Staph, epidermidis, account for 23%. This reflects the specifics of the infections seen in the intensive care unit of the institute and also the need for the strictest adherence to the rules of asepsis and antiseptics in the management of patients with EVD. Figure 3 shows the frequencies of identification of various pathogens.

Based on our center protocol of empiric antibiotic therapy, the initial systemic therapy of drainage-associated meningitis was carried out with a combination of carbapenems (meropenem, doripenem) and vancomycin in the form of prolonged infusion at the maximum dosage. Antimicrobial therapy was adjusted based on the antibiotic sensitivity of pathogens isolated from the CSF. In the absence of a pathogen from the CSF, the treatment was continued empirically. The duration of therapy depended on the rate of regression of inflammatory changes in the CSF and a decrease in markers of the systemic inflammatory response, and it averaged 10-14 days for gram-positive pathogens of drainage-associated meningitis and 14-21 days for gram-negative pathogens.

Determining the role of various factors in the outcomes of surgical treatment of patients with aneurysms in the acute period of SAH is difficult due to multiple, rarely independent, factors. A satisfactory outcome (mRS scores of 1 and 2) was observed in 24.7% (n=35) of the patients. Moderate and profound disability at the time of discharge was noted in 55.7% (n=79). Vegetative outcome at discharge was noted in 8.4% (n=12), and mortality occurred in 12.3% (n=15).

## Discussion

Indications for EVD in SAH patients

Indications for EVD in patients with aSAH remain unstandardized and subject to debate. Conditional indications for EVD implantation include ventriculomegaly, decreased wakefulness according to the Glasgow Coma Scale (score of 12 or less), H-H severity of II or more, H-H severity of III or more, and lack of command execution [9]. Some authors recommend EVD for all patients with aSAH, while others advise the installation of an EVD only when the clinical condition worsens [2].

Patients with SAH, in the early post-hemorrhagic period, have variations in their level of consciousness (from deep stunning to coma), and the etiology of neurological disorders cannot always be established [10]. Installing EVD for ICP monitoring and control is considered the first step in the treatment of such patients [6,11]. Lack of improvement after EVD and normalization of ICP might indicate other causes, such as seizures, deep sedation, metabolic disorders, and ischemic disorders. Despite the fact that the installation of EVD prior to occlusion or clipping of the aneurysm can improve the neurological status [12], this is not always associated with a favorable prognosis [13]. Also, many patients whose clinical condition does not improve after ventriculostomy show positive dynamics after surgery, when intracerebral hematomas (ICH) are removed during the operation, and the basal cisterns are debrided [13]. Ziai et al. have demonstrated that the installation of a ventricular drain in a group of patients with IVH could effectively monitor and reduce ICP [14]. This, in turn, is an indication for the implantation of EVD. In our opinion, the indications for EVD are an H-H score of III-V, as well as signs of ICHyp (IVH, ICH, severe ischemic disorders).

Preoperative EVD and the risk of repeated hemorrhage

The data on the risk of re-bleeding after ventriculostomy in patients with aneurysms remain ambiguous [9,10,15]. The placement of an EVD is thought to increase the risk of re-bleeding, especially when a drain is placed with a drainage threshold below 25 mmHg [9]. A sharp decrease in ICP during CSF drainage can lead to an increase in aneurysmal transmural pressure, which contributes to the repeated rupture of the aneurysm [15]. Paré et al. [16] have reported that in a group of 128 patients with aSAH, those with EVD had a higher frequency of re-bleeding than patients without EVD (30% versus 8.3%). However, some other studies have concluded that EVD is not associated with the risk of re-bleeding in patients with acute hydrocephalus after aSAH [9].

As a rule, patients who require EVD prior to surgery have a more severe clinical condition [6,16], which in turn is associated with independent factors of re-bleeding [5]. In addition, most studies did not take into account the following factor: the duration of ventriculostomy, especially the time during which the aneurysm remained unclipped or occluded [5,10]. This is significant because the risk of re-bleeding after the rupture of the aneurysm has a cumulative effect over time, regardless of EVD, with the highest frequency observed during the first few days after SAH [5,10]. Based on the experience at our department, the installation of an EVD should be combined with a one-stage surgical intervention on the aneurysm, which in turn does not affect the risk of re-bleeding against the background of EVD.

ICP monitoring

There is no such thing as an ideal ICP monitoring device [6]. The accuracy of ICP measurements is limited by the proximity of the device to the lesion and transducer position [17]. Monitoring of ICP through EVD is often associated with a high incidence of misalignment, dysfunction, and hence inaccurate ICP measurement [18]. Also, the installation of EVD is difficult in the presence of a significant mass effect or narrow ventricles. The use of neuronavigation improves the accuracy of implantation, especially in a situation with narrow or collapsed lateral ventricles, and can reduce the incidence of complications and malfunctions [8]. According to some authors, when EVD cannot be used to drain CSF, lumbar drainage can be used. However, in such cases, a separate ICP sensor is often required because lumbar drainage does not provide reliable monitoring [17]. Intraparenchymal ICP transducers are simple and easy to use, and their fiber optic or strain gauge catheters allow for accurate ICP monitoring. EVD and intraparenchymal sensors are equally sensitive to detecting ICP patterns, although the mean ICP might vary by 2-8 mmHg; for example, it may be lower or higher than the intraventricular pressure [19]. Installation of an EVD and an ICP sensor is associated with a certain risk of hemorrhagic complications (from 2 to 10%) [19]. Nevertheless, for severe patients [10,19], in line with some authors, we also recommend the simultaneous placement of an EVD and an intraparenchymal ICP sensor. Often, EVD must be used for a permanent discharge of CSF to reduce ICP; sometimes severe edema causes its dysfunction, which can lead to a sharp increase in ICP and deterioration of the patient’s condition. Therefore, for monitoring ICP in unconscious patients, we recommend using direct ICP monitoring with a parenchymal probe.

Terms of EVD removal

There is little available data on the timing of EVD removal. The question of the simultaneous removal versus gradual closure of the drainage with an assessment of the clinical condition has not been unequivocally resolved [20]. It is unclear whether a gradual reduction in CSF discharge has any advantage over stopping drainage at once. Klopfenstein et al. [21] conducted a prospective, randomized study to compare between simultaneous and gradual removal of EVD in patients with aSAH. A rapid removal of the EVD occurred within 24 hours with the immediate closure of the EVD, while gradual removal occurred over a 96-hour period with successive daily increases in the level of the EVD system with decreased drainage. The first group spent an average of more than 2.8 days in the intensive care unit (p=0.0002) and 2.4 days in the hospital (p=0.0314) compared to the group with rapid removal of the EVD. Based on the shorter hospital stay and the similar frequency of ventriculoperitoneal shunting in both groups, it was concluded that rapid removal from the EVD is as safe and potentially more practical than the gradual removal of the EVD [21]. In our department, for patients without obvious signs of hydrocephalus, the drainage is removed immediately after stopping drainage from eliminating the risk of liquorrhea through the wound channel along the drain.

Chronic hydrocephalus and the need for ventriculoperitoneal shunting

Many factors are associated with the development of chronic hydrocephalus after SAH [22]. In some cases, it is possible to determine the need for a shunt based on EVD. Chan et al. retrospectively reviewed 157 cases of SAH and identified multiple factors influencing the need for shunting operations, including the mean diameter of the third ventricle, bicaudate index, severity according to the H-H scale at the time of admission, and location of the aneurysm in the posterior parts of the circle of Willis. [23]. Long-term CSF drainage has been reported to be associated with an increased risk of chronic hydrocephalus after SAH [24]. However, a reliable relationship between these factors has not been established. According to our data, the incidence of VPS was 12.2%, which generally corresponds to the findings in the literature [22-24].

Most patients have CSF disorders (hygromas, ventriculomegaly) that do not have significant clinical manifestations. In such situations, we are not in a hurry to implant VPS, but we prefer to monitor the patient on an outpatient basis and, with the progression of clinical signs of hydrocephalus, we carry out diagnostics and treatment according to generally accepted recommendations [25]. In a situation where the patient, after EVD removal, demonstrates a deterioration in the cognitive status or level of wakefulness with obvious positive dynamics after unloading lumbar puncture, the VPS is performed one or several weeks later, provided that the CSF is completely debrided.

EVD-associated meningitis

According to the literature, the incidence of EVD-associated infection can be as high as 45% [18,25]. Different approaches to the diagnosis of EVD-associated infection in different studies make it difficult to generalize and compare the results [26]. Also, there is little research on EVD-associated meningitis that is specific to aSAH. The CSF profile (i.e., cell count, protein, and glucose levels) and culture should be monitored regularly. Several studies have demonstrated a direct relationship between the number of cells (cytosis) and the frequency of detection of pathogens during the microbiological examination of CSF [27]. Diagnosis of meningitis is a difficult clinical task, as there are no established criteria to confirm the presence of postoperative meningitis, or to exclude this complication with confidence [27]. Data on the cellular composition, as well as biochemical and microbiological studies of CSF, should be assessed in a complex and dynamic way. Additional laboratory tests are as follows: the study of procalcitonin in the CSF and blood serum and polymerase chain reaction (PCR) of the CSF to search for the DNA of the main pathogens causing meningitis [27].

Some studies have suggested that CSF sampling increases the risk of infection and that CSF samples should only be taken if there is clinical suspicion of infection [26]. A meta-analysis of 160 patients in seven studies showed that randomly collected daily CSF samples did not detect bacterial colonization (infection) more often than samples from patients with clinical signs of meningitis [28]. Although the administration of prophylactic systemic antibiotics against the background of EVD is common practice in many settings, their effectiveness in preventing EVD-associated meningitis has not been proven [29].

In our opinion, without clinical and laboratory signs of an infectious process, the initiation of antibiotic therapy against the background of EVD is not indicated. Among the risk factors that significantly increase the incidence of drainage-associated meningitis, one can single out those that are directly related to the installation and use of EVD (duration of drainage, combination with liquorrhea) and others associated with specific conditions in neurosurgical patients (craniotomy, infectious lesions of the soft tissues of the head, and liquorrhea) [29]. As our study has shown, despite the more frequent use of EVD in the second period, the risk of infectious complications decreased for the prevention of risk factors according to the protocol adopted at the BNC; nevertheless, further research is needed regarding the preventive measures [30].

## Conclusions

Based on our findings, EVD ensured effective monitoring and reduction of ICP. EVD was associated with a relatively low risk of infectious, liquorodynamic, and hemorrhagic complications and did not worsen outcomes when used in patients with aSAH. We recommend that all patients in the acute stage of SAH with H-H severity of III-V receive EVD immediately before surgery.
